# Restoring the Ocular Integrity of Perforated Corneal Ulcer Using Living Surgical Donor Tissues Derived From Keratoplasty in the COVID-19 Pandemic

**DOI:** 10.7759/cureus.53607

**Published:** 2024-02-05

**Authors:** Divya Kumari, Rakhi Kusumesh, Bibhuti Sinha, Nilesh Mohan, Mohamed Asif

**Affiliations:** 1 Ophthalmology, Regional Institute of Ophthalmology, Indira Gandhi Institute of Medical Sciences, Patna, Patna, IND; 2 Ophthalmology, Eye7 Chaudhary Eye Centre, Delhi, IND

**Keywords:** covid-19, fungal, therapeutic penetrating keratoplasty, pseudophakic bullous keratopathy, infectious keratitis

## Abstract

The coronavirus disease 2019 (COVID-19) caused an unprecedented crisis for corneal surgeons who were forced to strategize for an acute shortage of tissues. Here, we report the initial clinical outcomes of utilizing host corneal buttons derived from optical penetrating keratoplasties of pseudophakic bullous keratoplasty (PBK) patients. Two patients presented to our department with a perforated fungal corneal ulcer in one eye during the COVID-19 pandemic. One eye of each of the patients was operated on with non-vascularized host cut tissues preserved in glycerin. The tissues were secured using 10-0 nylon sutures. Good anatomical integrity was achieved in both eyes. An optical penetrating keratoplasty (PK) was done in both eyes after one year for visual rehabilitation, with a final visual acuity of 20/120 and 20/80, respectively, at six months. In conclusion, therapeutic PK using host tissues obtained from the recipients of optical PK is a safe and effective option to restore ocular integrity during a shortage of fresh or glycerol-preserved corneas. However, optical PK is required for the final visual rehabilitation.

## Introduction

Therapeutic penetrating keratoplasty (TPK) is a surgical procedure, the purpose of which is either to restore globe integrity or to resolve infectious or inflammatory keratitis nonresponsive to medical treatment. During the lockdown, the number of donor corneas collected and thus the emergency therapeutic keratoplasties performed decreased significantly because of travel restrictions and lockdown [1-3]. In addition, tissues from other eye banks could not be transported. Many ophthalmologists resorted to the use of glycerol-preserved corneas [4] or scleral patch grafts [5] for therapeutic purposes. Other possible alternatives for emergency use were small incision lenticule extraction (SMILE)-derived lenticule patch grafts [6], a bandage contact lens with glue, amniotic membrane transplantation(AMT) [7], and umbilical cord patch [8].

Goto et al. [9] have reported the use of cornea obtained from keratoconus eyes for therapeutic lamellar keratoplasty or keratoepithelioplasty. The primary objective of our study was to explore alternative surgical methods during tissue shortages. In our cases, we anticipated tissue scarcity due to the coronavirus disease 2019 (COVID-19) pandemic and promptly preserved four host tissues derived from optical penetrating keratoplasty (PK) for pseudophakic bullous keratopathy (PBK) at 4°C in a sterile glass bottle with a sterile glycerin solution. We used two of these tissues for therapeutic purposes. Written informed consent was taken from donors who were also the recipients of the optical graft.

## Case presentation

Case 1

**Figure 1:**
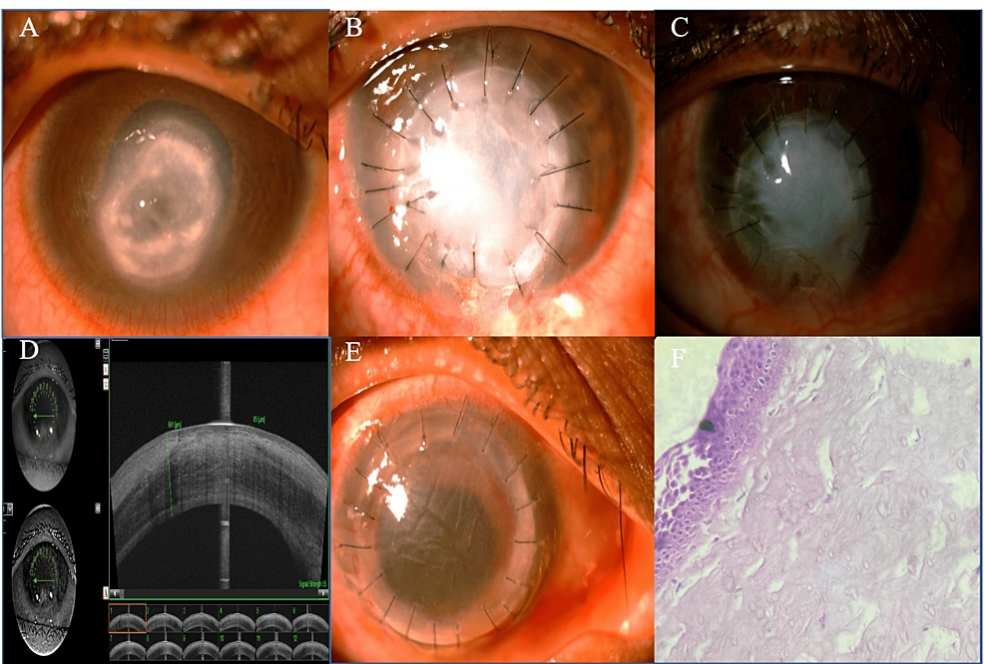
A) Diffuse slit lamp view of patient 1 before therapeutic PK, showing a perforated corneal ulcer with pseudo-cornea with an inferior shallow anterior chamber. B) Postoperative day 1 of therapeutic penetrating keratoplasty with the host tissue. C) At one year after therapeutic penetrating keratoplasty. D) Anterior segment optical coherence tomography (ASOCT) image of the eye of patient 1 showing a thick epithelialized graft. E) On postoperative day one after optical PK. F) Histopathology showing a stratified squamous non-keratinized five-layer epithelium (haematoxylin and eosin staining x40).

In April 2020, in the Cornea Clinic of the Department of Ophthalmogy, Indira Gandhi Institute of Medical Sciences (IGIMS), Patna, a 28-year-old female presented to us with redness, discharge, and decreased vision in the right eye (RE) following trauma with a wooden stick of six days' duration (Table 1). She had no systemic illness. On ocular examination, she had visual acuity of hand motions with accurate projection of rays in the RE and 20/20 in the left eye (LE). A perforated corneal ulcer with the greatest diameter of 5.1 x 4.2 mm with 0.5 mm diffuse, full-thickness, stromal infiltrate in the surrounding stroma. The anterior chamber (AC) was shallow inferiorly (Fig. 1A). B-scan ultrasonography of the RE was anechoic. The LE was normal. Potassium hydroxide (KOH) wet mount of corneal scraping showed filamentous fungi with acute angle branching. Culture on Sabouraud dextrose agar grew *Aspergillus flavus*. The patient was started on 5% natamycin eyedrop one hourly, homatropine 2% four times a day, moxifloxacin 0.5% six times a day, and 200 mg ketoconazole tablet twice daily. TPK was performed with a glycerol-preserved, non-vascularized host tissue, obtained from the optical PK of the PBK patient on the 23rd day of its storage. We performed standard blood infection tests preoperatively in all the patients undergoing surgery at our institute; hence, the living surgical tissue donors were tested preoperatively before receiving the optical graft and, at the same time, their host cut tissues were preserved. At the time of surgery, the donor corneal button was soaked for 10 min in a sterile basal salt solution (BSS) to leach out residual glycerin. A portion (0.5 mm) of the normal cornea surrounding the infiltrate was trephined. A peripheral iridectomy with an intracameral wash of voriconazole (50 mg/0.1 mL) was done. The size of the graft was 7.5 mm. The donor button was oversized by 0.5 mm. On the first postoperative day, the graft clarity was zero, the graft host junction was well-apposed, and the anterior chamber was formed (Fig. 1B). The patient was maintained on the same medications as advised preoperatively.

**Table 1:** Summarized clinical findings, laboratory findings, and outcomes of host tissue keratoplasy

Charecteristics	Case 1	Case 2
Age (years)	28	68
Gender	Female	Female
Details of trauma	Wooden stick trauma	None
Symptoms	Redness, discharge, and decreased vision for six days' duration	Redness, pain, photophobia, and decreased vision for one-month duration
Corrected distance visual acuity (CDVA)	Hand motions with the projection of rays accurate in all quadrants	Perception of light with the projection of rays accurate in all quadrants
Laterality	Right eye	Left eye
Details of keratitis	Perforated corneal ulcer of greatest diameter of 5.1 x 4.2 mm with 0.5 mm diffuse full-thickness, stromal infiltrate in the surrounding corneal stroma. Anterior chamber shallow inferiorly B-scan ultrasonography	Perforated corneal ulcer of size 4.7 x 4.5 mm, Descemet membrane folds, and shallow anterior chamber. The crystalline lens was cataractous.
Laboratory test	Culture on Sabourad dextrose agar (SDA) grew *Aspergillus flavus.*	Culture on SDA grew *Aspergillus flavus.*
Medical management	Eyedrops natamycin 5%, moxifloxacin 0.5%, homatropine 2%, tablet ketoconazole 200 mg twice daily	Eyedrops natamycin 5%, moxifloxacin 0.5%, homatropine 2%, tablet ketoconazole 200 mg twice daily
Surgical management	Therapeutic penetrating keratoplasty with a host tissue of graft size 7.5 mm	Therapeutic penetrating keratoplasty with a host tissue of graft size 7.5 mm
Optical penetrating keratoplasty done	Yes after one year	Optical triple procedure after 14 months
Visual outcome after optical graft	20/200 at the six-month postoperative visit	20/80 at the six-month postoperative visit

There was no recurrence of infection in the postoperative period. After one month, the patient was followed up through telemedicine services. The graft was completely opaque with inferior conjunctivalization at one year (Fig. 1C). Anterior segment optical coherence tomography (ASOCT) was done with the DRI OCT Triton (Topcon, Tokyo, Japan) preoperatively, which was suggestive of a thick epithelialized graft with an apposed graft-host junction (Fig. 1D). The graft thickness was 991 microns and epithelial thickness was 85 microns. An optical PK was performed after one year (Fig. 1E). The final visual acuity at the six-month postoperative visit was 20/120. The histopathological examination (HPE) showed a thick stratified squamous non-keratinized epithelium and edematous stroma (Fig. 1F).

Case 2

**Figure 2:**
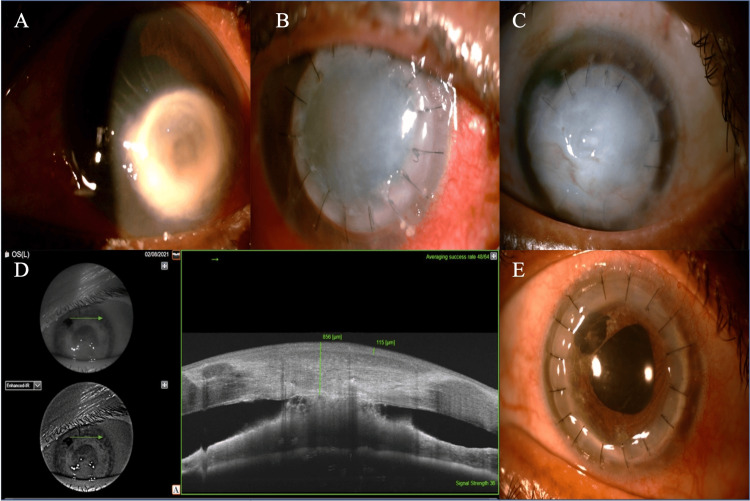
A) Preoperative slit lamp view of patient 2 showing a perforated corneal ulcer. B) Day one post therapeutic PK showing a well-apposed wound. C) One year post therapeutic PK showing an opaque graft. D) Anterior segment optical coherence tomography (ASOCT) picture of patient 2 showing a thick epithelialized graft with peripheral anterior synechiae (PAS) at the graft-host junction superiorly. E) After optical PK.

In April 2020, a 68-year-old female presented with complaints of redness, pain, photophobia, and decreased vision in LE, for a duration of one month. The best corrected visual acuity was 20/20 in the RE and hand motions in the LE. On slit-lamp examination, the patient had an LE perforated corneal ulcer of size 4.7 x 4.5 mm, with Descemet membrane folds and a shallow anterior chamber (Fig. 2A). B-scan ultrasonography of the LE was anechoic. The RE had an immature senile cataract with a normal posterior segment. KOH wet mount of corneal scraping showed filamentous fungi with acute angle branching. Culture on Sabouraud dextrose agar grew *Aspergillus flavus*. TPK was performed with host tissue using the same preservation method. The tissue was utilized on day 31st of its storage in glycerin. On the first postoperative day, graft clarity was one plus [10], and the wound was well apposed. No exudate or hypopyon was noted. AC details were very faintly seen (Fig. 2B). Postoperatively, the patient was started on 5% natamycin eyedrop one hourly, homatropine 2% four times a day, moxifloxacin 0.5% six times a day, and 200 mg ketoconazole tablet twice daily. No recurrence of infection was noted during the follow-up. Due to the lockdown, he was followed up through telemedicine. After the easing lockdown restrictions, the visual acuity in the LE was a perception of light with the projection of rays accurate in all quadrants. The graft clarity was zero [10], with deep vascularization at 4 and 5 o'clock positions. Partial suture removal was done (Fig. 2C). ASOCT at one year was suggestive of a thick epithelialized graft (856 microns), with peripheral anterior synechiae (PAS) superiorly, at the graft-host junction (Fig. 2D). An optical triple procedure was done at the 14th month when the donor tissue became available (Fig. 2E). The patient’s final visual acuity was 20/80 at six months post optical penetrating keratoplasty. 

## Discussion

The COVID-19 pandemic caused an unprecedented crisis for corneal surgeons who were forced to strategize [1,11] and provide care in situations of acute imbalance of demand and supply of tissues. In our cases, we used devitalized tissue with almost no endothelial cell count, so the grafts were oedematous, thick, and opaque. We assumed that these patients may continue to have infections similar to other therapeutic penetrating keratoplasty cases and have suboptimal vision in the follow-up and that they would eventually require subsequent optical penetrating keratoplasty. We took proper consent explaining these risks.

Due to difficulty in the visualization of anterior chamber details, the ongoing infectious process was indirectly assessed using a combination of clinical signs and symptoms, ultrasonography, and visualization from the peripheral clear corneal rim of the recipient. Even though the donor corneal buttons were oversized by 0.5 mm, angle distortion and anterior chamber collapse were anticipated because thick, devitalized tissues were being used. Hence, the patients were started on prophylactic antiglaucoma drops through telemedicine.

Both cases had good anatomical integrity and no recurrence of infection, secondary infection, or persistent epithelial defects, till two years from the initial presentation to our department. On histopathological examination, the epithelium was found to be thick and hypertrophied, stratified squamous, and non-keratinized with stromal edema. In addition, the stromal edema and corneal thickness can be decreased by collagen cross-linking of the donor tissue preoperatively.

## Conclusions

TPK using host tissues obtained from recipients of optical PK, where corneal dystrophies and infections have been ruled out, is a safe and effective option in cases of emergency to temporarily restore ocular integrity when other options like fresh or glycerine-preserved tissues are not available or when the defect is too large to be closed with bandage contact lens or amniotic membrane or tenon’s patch graft. When the infection is cleared, however, an optical PK using tissues with a viable endothelium is required for visual rehabilitation. This case report provides detailed observations on a few subjects, which may not be sufficient to make broad generalizations; hence, larger studies are required.

## References

[1] Agarwal R, Sharma N, Patil A, Thakur H, Saxena R, Kumar A (2020). Impact of COVID-19 pandemic, national lockdown, and unlocking on an apex tertiary care ophthalmic institute. Indian J Ophthalmol.

[2] Thuret G, Courrier E, Poinard S (2022). One threat, different answers: the impact of COVID-19 pandemic on cornea donation and donor selection across Europe. Br J Ophthalmol.

[3] AlMutlak M, Li JY, Bin Helayel H, Fairaq R (2021). Future of corneal donation and transplantation: insights from the COVID-19 pandemic. Cornea.

[4] Gupta N, Dhasmana R, Maitreya A, Badahur H (2020). Glycerol-preserved corneal tissue in emergency corneal transplantation: an alternative for fresh corneal tissue in COVID-19 crisis. Indian J Ophthalmol.

[5] Siva Jyothi EL, Gopala Krishna O, Lakshmana Rao P, Raja Sekhar P (2021). Case series of scleral patch grafts during COVID-19 pandemic. Indian J Ophthalmol.

[6] Yang H, Zhou Y, Zhao H, Xue J, Jiang Q (2020). Application of the SMILE-derived lenticule in therapeutic keratoplasty. Int Ophthalmol.

[7] Gicquel JJ, Bejjani RA, Ellies P, Mercié M, Dighiero P (2007). Amniotic membrane transplantation in severe bacterial keratitis. Cornea.

[8] Xie HT, Zhao D, Liu Y, Zhang MC (2017). Umbilical cord patch transplantation for corneal perforations and descemetoceles. J Ophthalmol.

[9] Goto S (1999). Therapeutic keratoplasty using preserved corneas from keratoconus eyes. Jpn J Ophthalmol.

[10] Sihota R, Sharma N, Panda A, Aggarwal HC, Singh R (1998). Post-penetrating keratoplasty glaucoma: risk factors, management and visual outcome. Aust N Z J Ophthalmol.

[11] Roy A, Das S, Chaurasia S, Fernandes M, Murthy S (2020). Corneal transplantation and eye banking practices during COVID-19-related lockdown period in India from a network of tertiary eye care centers. Indian J Ophthalmol.

